# Icariin Regulates the Bidirectional Differentiation of Bone Marrow Mesenchymal Stem Cells through Canonical Wnt Signaling Pathway

**DOI:** 10.1155/2017/8085325

**Published:** 2017-12-27

**Authors:** Jun-ming Huang, Yuan Bao, Wei Xiang, Xing-zhi Jing, Jia-chao Guo, Xu-dong Yao, Rui Wang, Feng-jin Guo

**Affiliations:** Department of Orthopedics, Tongji Hospital, Tongji Medical College, Huazhong University of Science and Technology, Wuhan, Hubei 430030, China

## Abstract

Fat infiltration within the bone marrow is easily observed in some postmenopausal women. Those fats are mainly derived from bone marrow mesenchymal stem cells (BMMSCs). The increment of adipocytes derived from BMMSCs leads to decreased osteoblasts derived from BMMSCs, so the bidirectional differentiation of BMMSCs significantly contributes to osteoporosis. Icariin is the main extractive of Herba Epimedii which is widely used in traditional Chinese medicine. In this experiment, we investigated the effect of icariin on the bidirectional differentiation of BMMSCs through quantitative real-time PCR, immunofluorescence, western blot, and tissue sections in vitro and in vivo. We found that icariin obviously promotes osteogenesis and inhibits adipogenesis through detecting staining and gene expression. Micro-CT analysis showed that icariin treatment alleviated the loss of cancellous bone of the distal femur in ovariectomized (OVX) mice. H&E staining analysis showed that icariin-treated OVX mice obtained higher bone mass and fewer bone marrow lipid droplets than OVX mice. Western blot and immunofluorescence showed that icariin regulates the bidirectional differentiation of BMMSCs via canonical Wnt signaling. This study demonstrates that icariin exerts its antiosteoporotic effect by regulating the bidirectional differentiation of BMMSCs through the canonical Wnt signaling pathway.

## 1. Introduction

Osteoporosis (OP) is a prevalent systemic metabolic bone disease that is featured by microarchitectural deterioration, low bone mass, and increased risk of fractures, which results from the unbalance between bone loss and bone formation. The presence of fat infiltration within the marrow cavity is a symbol of aging and a consequence of osteoporosis, particularly in menopausal women [1]. One possible source of lipid formation within the bone marrow is the disrupted balance between osteogenesis and adipogenesis of BMMSCs, and the latter assumes predominance [2, 3]. BMMSCs, the most important MSCs in the bone morrow, are self-renewable, multipotent stem cells with the capacity to differentiate into cartilage, bone, and adipose tissue [4–6]. The contributing factors of the formation of osteoblasts are recognized as an inhibitor for the maturation of adipocytes and vice versa [7–9]. Recent studies showed that various chemical compounds have the ability to regulate survival and differentiation of stem cells, such as bioflavonoids, vitamin E, vitamin C, and carotenoids [10–15]. The increased proportion of adipocytes in the bone marrow consequently induces the apoptosis of osteoblasts and proliferation of osteoclasts and suppresses the function of osteoblasts, which contribute to the progress of osteoporosis [16, 17]. Thus, regulation of osteogenesis and adipogenesis of BMMSCs seems to be a potential therapeutic strategy for osteoporosis.

Icariin (ICA, C_33_H_40_O_15_, molecular weight: 676.67), a natural flavonoid glycoside isolated from Herba Epimedii, is the main active compound responsible for its biological effect. In traditional Chinese medicine (TCM), its tonic, aphrodisiac, and antiosteoporotic effectiveness were recognized for thousands of years in Asian countries, principally China, Japan, and Korea. In a clinic study, bone mineral density (BMD) and bone strength of postmenopausal women were significantly improved via icariin treatment for 24 months [18]. Several studies had shown that, during bone formation, maturation and mineralization of osteoblasts and osteoblastic differentiation of BMMSCs could be improved and enhanced by icariin treatment [19]. During bone resorption, maturation and formation of osteoclasts, mediated by the inflammatory mediums and cytokines, could be suppressed by icariin [19]. Several bone metabolism-related signaling molecules such as Notch, extracellular signal-regulated kinase (ERK), c-Jun N-terminal kinase (JNK), hypoxia-inducible transcription factor 1*α* (HIF1*α*), and RhoA could be regulated by icariin [20–24]. Other studies also reported that icariin exerts beneficial effects on the skeletal system. Icariin could increase angiogenesis through promoting endothelial cell migration, proliferation, and tubulogenesis, and then the newborn blood vessel supports blood supply for bone formation [25]. In a rotator cuff tear model, icariin group could stimulate expression of Collagen I and Collagen II compared with control group at 2 weeks and 4 weeks, and more VEGF-positive cells were detected in the icariin group than in the control group [26].

In other biological processes, icariin was reported as an agonist to peroxisome proliferator activated receptor-*α* (PPAR*α*), with a pivotal role in the regulation of lipid metabolism and fatty acid oxidation, which is beneficial in protecting against metabolic disorders associated with type II diabetes and obesity [27]. In another study, icariin could improve diet-induced obesity and hyperlipidemia and alleviate insulin resistance through targeting sterol regulatory element-binding proteins (SREBPs) and suppressing their activation [28]. Furthermore, icariin could suppress lipid accumulation in a model of Graves' orbitopathy [29].

The Wnt signaling pathway is an ancient and evolutionally conserved pathway which plays a fundamental role in cell migration, cell polarity, cell fate determination, and organogenesis during embryonic development and tissue homeostasis [30, 31]. A decade ago, four groups of patients with variation in bone density, high or low bone mass, were identified to associate with the mutations in canonical WNT signaling. Two of the four mutations causing totally altered bone mass and density are found to be LRP5, responsible for the Wnt coreceptor low density lipoprotein receptor-related protein 5. In osteoporosis-pseudoglioma syndrome (OPPG), loss-off-function mutation in this gene is associated with low bone mass, and in some healthy patients, high bone mass was associated with gain-of-function mutation in the same gene [32]. The other two mutations affected the expression of a common gene, SOST, encoding protein sclerostin, secreted primarily from osteocytes, which binds LRP5 and the related LRP4 and LRP6 receptors and functions as an antagonist in Wnt signaling [33]. Lack of sclerostin expression in bone was found to be the cause for sclerosteosis and Van Buchem disease with a symptom of high bone mass [34]. Some research reported that adipogenesis of BMMSCs was under the regulation of Wnt signaling pathway and activation of *β*-catenin leading to inhibitory adipogenesis [35].

Based on the above evidence, our hypothesis was that icariin can direct differentiation toward osteoblast lineage and away from adipocyte lineage in differentiation of BMMSCs. In this study, we aim to detect whether icariin regulates adipogenic and osteogenic differentiation of BMSCs in vitro and in vivo and identify whether the Wnt signaling pathway plays a fundamental role in osteoadipogenic differentiation of BMMSCs.

## 2. Materials and Methods

### 2.1. Reagents

Icariin was purchased from Cayman (Ann Arbor, MI, USA). Fetal bovine serum (FBS) was purchased from Gibco Life Technologies (Grand Island, NY, USA). Dulbecco's modified Eagle's medium (DMEM), penicillin, and streptomycin were purchased from Hyclone (Hyclone, Waltham, MA, USA). The Cell Counting Kit-8 (CCK-8) was purchased from Dojindo (Kumamoto, Japan). Adipogenic and osteogenic differentiation media of rat bone marrow mesenchymal stem cells were purchased from Cyagen Biosciences Inc. (Santa Clara, CA 95050, USA). The total RNA extraction kit was purchased from OMEGA. The ReverTra Ace kit and SYBR Green Master Mix were purchased from TOYOBO (Tokyo, Japan). DKK-1, inhibitor of canonical Wnt signaling pathway, was purchased from R&D Systems (Minneapolis, MN, USA). The following antibodies used in the experiment were purchased from Cell Signaling Technology (Beverly, MA, USA): phospho-GSK-3*β* (#8213), *β*-catenin (#9562), Non-phospho (Active) *β*-Catenin (#8814), and GAPDH (#2118). Other reagents were of the highest commercial grade and were purchased from Sigma Chemical (St. Louis, MO, USA)

### 2.2. BMMSCs Culture

Briefly, rat BMMSCs used in the experiment were isolated from 4-week-old male Sprague-Dawley rats purchased from the Experimental Animal Center of Tongji Medical College, Huazhong University of Science and Technology (Wuhan, China), and expanded in accordance with published techniques [36]. The animal procedures were consistent with the regulations of the Ethics Committee on Animal Experimentation of Tongji Medical College, Huazhong University of Science and Technology (Wuhan, China). Isolated cells were maintained in DMEM consisting of 10% FBS, 2 mM glutamine, 100 U/ml penicillin, and 100 mg/ml streptomycin and were then incubated at 37°C in a humidified atmosphere of 95% air and 5% CO_2_. When cells became confluent, cells were trypsinized with 0.5% trypsin-ethylenediaminetetraacetic acid and passaged at a ratio of 1 : 3. BMMSCs of passage 2-3 were used for the following experiment. In the differentiation experiment of BMMSCs, cells were cultured in adipogenic and osteogenic differentiation media for 21 days following the operation instruction by changing the medium every 2 days. Icariin (1 *μ*M) and DKK-1 (50 ng/ml) were used to detect the function of icariin in osteogenesis and adipogenesis differentiation of BMMSCs.

### 2.3. CCK-8 Proliferation Assay

BMMSCs were seeded in 96-well plates at a density of 1 × 10^3^ cells/well. Each group had five subwells. After 12-hour incubation, cells were treated with icariin at 0.001, 0.01, 0.1, 1, and 10 *μ*M, respectively. Cell proliferation was carried out by the CCK-8 kit (Dojindo, Japan) in a culture after cultivating for 1 day, 3 days, 5 days, and 7 days, respectively. In brief, 10 *μ*L CCK-8 solution was added to each well and incubated in the dark at 37°C with 5% CO_2_ for 1.5 h, and then the absorbance was measured at 450 nm by an ELX800 absorbance microplate reader (BioTek, Vermont, USA).

### 2.4. Total RNA Extraction and Quantitative Real-Time RT-PCR

Total RNA was extracted by total RNA extraction kit, OMEGA. The purity and concentration of the RNA samples were determined by a spectrophotometer (Thermo Fisher Scientific, USA). The ReverTra Ace kit was used for the synthesis of first-strand cDNA from 3 *μ*g RNA and quantitative real-time PCR was carried out with CFX96 (Bio-Rad, CA, USA) and SYBR Green Master Mix. The relative expression of Runx2, PPAR*γ*, ALP, Collagen I, adipsin, and Fabp4 was quantified in this system. Primers were purchased from Sangon Biotech (Shanghai, China), and their sequences are shown in Table 1. The system of reactions consisted of denaturation at 95°C for 30 s followed by 40 cycles of 94°C for 5 s and 60°C for 35 s. Relative expression of gene-specific products was analyzed using the comparative Ct (2^−ΔΔCt^) method and normalized to the reference gene glyceraldehyde-3-phosphate dehydrogenase (GAPDH). Each sample was detected in triplicate.

### 2.5. Alizarin Red S Staining and Oil Red O Staining

In osteogenic differentiation, BMMSCs were cultivated at 2 × 10^4^ cells/cm^2^ on six-well plates and incubated for 21 days in an osteogenic induction medium consisting of SD rat BMMSCs osteogenic differentiation basal medium (175 mL), fetal bovine serum (20 mL), penicillin/streptomycin (2 mL), glutamine (2 mL), ascorbate (400 *μ*L), *β*-glycerophosphate (2 mL), and dexamethasone (20 *μ*L).

In adipogenic differentiation, BMMSCs were cultivated at 2 × 10^4^ cells/cm^2^ on six-well plates and incubated in an adipogenic induction medium consisting of SD rat BMMSCs adipogenic differentiation basal medium A (175 mL), fetal bovine serum (20 mL), insulin (400 *μ*L), glutamine (2 mL), rosiglitazone (200 *μ*L), dexamethasone (200 *μ*L), 3-isobutyl-1-methylxanthine (200 *μ*L), and penicillin/streptomycin (2 mL) for 3 days, followed by 1 day with an adipogenic maintenance medium consisting of SD rat BMMSCs adipogenic differentiation basal medium B (175 mL), fetal bovine serum (20 mL), insulin (400 *μ*L), glutamine (2 mL), and penicillin/streptomycin (2 mL). When both steps were repeated five cycles, cells were ready for Oil Red O staining.

Icariin (1 *μ*M) and DKK-1 (50 ng/ml) were treated, respectively, and jointly into induction medium every 2 days. When cells were ready for Alizarin Red S staining and Oil Red O staining, samples were washed with PBS twice and fixed with 4% paraformaldehyde for 30 min at room temperature. In Alizarin Red S staining, fixed samples were treated with 1 mL Alizarin Red S staining solution for 3–5 minutes. In Oil Red O staining, fixed samples were treated with 1 mL of Oil Red O staining solution for 30 minutes. Both samples were gently rinsed by tridistilled water, followed by investigation under a light microscope and capturing of images.

### 2.6. Alkaline Phosphatase (ALP) Staining

BMMSCs were cultivated at 2 × 10^5^ cells/cm^2^ on six-well plates and then treated with icariin (1 *μ*M) and DKK-1 (50 ng/ml), respectively and jointly, in an osteogenic medium. After 7 days of induction, leukocyte alkaline phosphatase (ALP) kits (Sigma, USA) were used for Al staining. Samples were washed with PBS twice, fixed in 4% paraformaldehyde for 10 min, and stained with a BCIP/NBT alkaline phosphatase color development kit (Beyotime, Shanghai, China) under protection from direct light at 37°C. A light microscope was used for observing and taking photos.

### 2.7. Western Blot Assay

Samples were lysed via the protein extraction reagent RIPA (BOSTER, Wuhan, China) supplemented with 1 mM PMSF and phosphatase inhibitor cocktail (BOSTER, Wuhan, China). The concentration of samples was measured through a BCA protein assay kit (BOSTER, Wuhan, China). Prior to loading, a 5x reducing sample loading buffer (BOSTER, Wuhan, China) was added to all the protein samples and denatured at 95°C for 5 min. An equivalent amount of protein, 20 *μ*g of every sample, was resolved by 10% SDS-PAGE gel and transferred to PVDF membranes (Millipore, Billerica, MA, USA). Membranes were blocked and incubated with individual primary antibodies and then were washed and incubated with secondary antibodies conjugated with horseradish peroxidase (BOSTER, Wuhan, China; dilution: 1 : 5000). The immunoreactive proteins were detected by enhanced chemiluminescence (BOSTER, Wuhan, China) and the bands of protein were captured through ChemiDoc™ XRS+ system with Image Lab™ software (Bio-Rad, CA, USA).

### 2.8. Immunofluorescence

BMMSCs were seeded on aseptic 1 cm diameter disks in a 24-well plate with density of 2 × 10^4^ cells/disc for 4 days. Samples were washed with PBS and fixed in 4% paraformaldehyde for 15 min at room temperature. The cells were washed with PBS three times and blocked with PBS containing 5% BSA for 1 h. After being blocked with BSA, samples were incubated with primary antibody *β*-catenin (1 : 200) (CST, MA, USA) overnight at 4°C. On the following day, samples were washed with PBS three times and incubated with the goat anti-rabbit Cy3-labeled secondary antibody (1 : 500) (BOSTER, Wuhan, China) which was diluted in a blocking solution for 1 h at room temperature. Samples were further counterstained with DAPI (1 : 500) (BOSTER, Wuhan, China) and phalloidin (1 : 100) (Beyotime, Shanghai, China). Inverted fluorescent microscope (Eclipse Ti, Nikon, Tokyo, Japan) was used to capture fluorescent images.

### 2.9. Ovariectomized Mouse Model

Briefly, three-month-old female C57/BL6 mice (22 ± 1 g) were purchased from the Experimental Animal Center of Tongji Medical College (Wuhan, China) and were randomly divided into three groups (*n* = 10 mice per group): sham-operated mice (SHAM), ovariectomized (OVX) mice treated with vehicle (OVX), and OVX mice treated with icariin (OVX + ICA). All experimental procedures on the animals were in accord with the Ethics Committee on Animal Experimentation of Tongji Medical College, Huazhong University of Science and Technology (Wuhan, China). The ovariectomized model was accomplished by removal of bilateral ovaries through a dorsal approach and sham surgery was accomplished by detecting the bilateral ovaries. On the following day after operation, mice were treated orally with icariin (25 mg/kg) or vehicle daily. After 6 weeks of intervention, animals of the three groups were euthanized with excess amounts of pentobarbitone and the femurs were collected.

#### 2.9.1. Histologic Analysis

Femur samples were in decalcification in 10% tetrasodium-EDTA aqueous solution at 4°C for 1 week. Paraffin-embedded sections (5 mm) from each femur sample were prepared for hematoxylin and eosin (H&E) staining. The cancellous bone and lipid droplets in the region below the metaphysis were observed microscopically. The trabecular bone density and lipid droplets area of interest were measured and compared in H&E-stained sections.

#### 2.9.2. Microcomputed Tomography (*μ*-CT) Scanning and Analysis

The bone samples of the three groups were scanned through a Scanco VivaCT 40 instrument (Scanco, Brüttisellen, Switzerland). The bone samples were scanned in a setting of 100 kV and 98 *μ*A and the thickness of serial tomographic images was 10.5 *μ*m. Subsequently, the obtained outcomes of bone structure were analyzed as previously reported [37]. The constant threshold for trabecular bone was 220 for segmenting bone from the bone marrow. The region of interest (ROI) in trabecular bone was selected from 10 slices (105 *μ*m) from the metaphysis of the distal femur, and the length range was 150 slices (1575 *μ*m). Four morphometry parameters, that is, bone volume/tissue volume (BV/TV), trabecular number (Tb.N), trabecular thickness (Tb.Th), and trabecular separation (Tb.Sp), were quantitatively measured using software compatible with the *μ*-CT system. Nomenclature and abbreviations of 3D-*μ*CT parameters are in accord with the recommendations of the American Society of Bone and Mineral Research [38].

#### 2.9.3. Statistical Analysis

The quantitative data were expressed as mean ± SD from three independent experiments. Student's* t*-test was selected for assessing the significance of differences between two groups. One-way ANOVA (SPSS, v.19.0; Chicago, IL, USA) was selected for assessing the significance of differences among three or more groups. The difference was denoted as statistically significant at *P* < 0.05.

## 3. Results

### 3.1. Effects of Icariin on Bone Loss in OVX Mice

**Figure 1:**
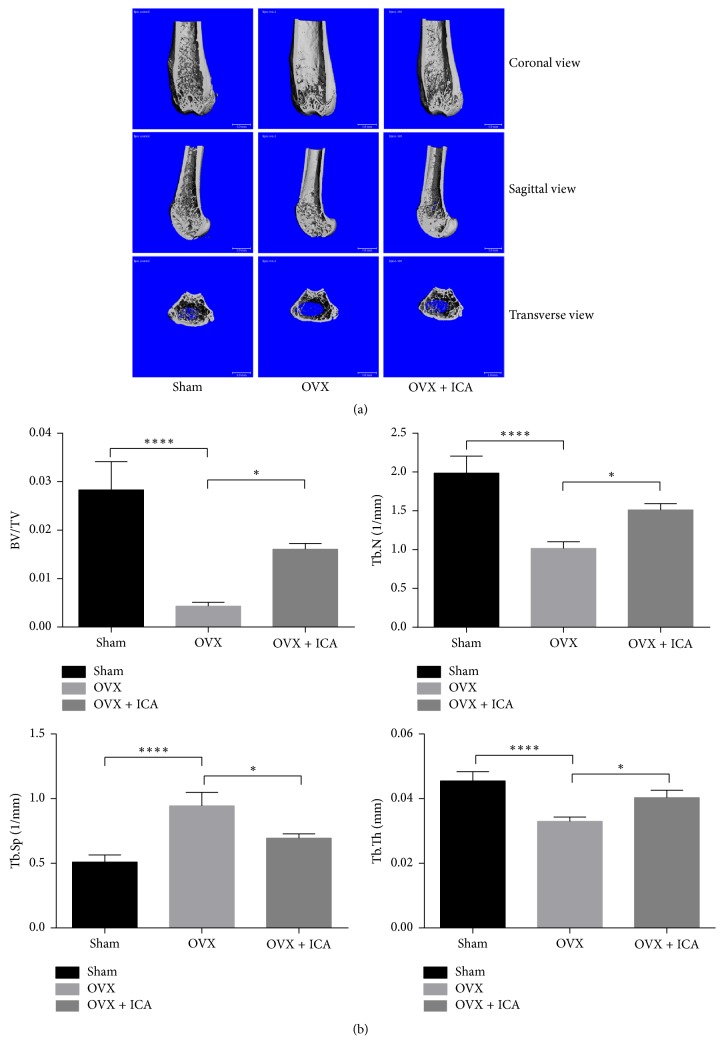
Icariin inhibits OVX-induced bone loss. (a) Images of the distal femurs were obtained after 6-week intervention (top, coronal view of the metaphyseal region; middle, sagittal view; bottom, transverse view). (b) Analysis of the parameter of the three-dimensional trabecular structure of the distal femur: BV/TV: trabecular bone volume/tissue volume; Tb.N: trabecular number; Tb.Sp: trabecular separation; Tb.Th: trabecular thickness. Data represented as mean ± SD, *n* = 10. ^*∗*^*P* < 0.05; ^*∗∗∗∗*^*P* < 0.0001.

**Figure 2:**
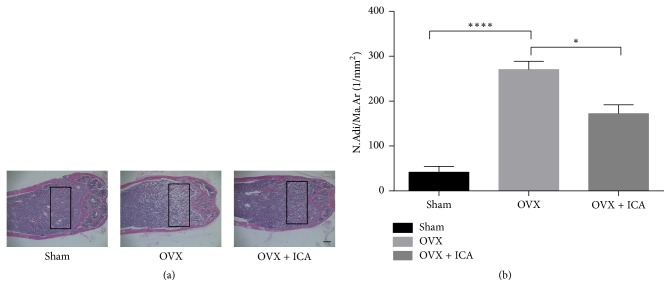
Icariin inhibits angiogenesis in VOX mouse. (a) Femurs were fixed and then embedded in paraffin. Scale bar: 40x magnification. Images stained with H&E in the distal femurs were obtained. (b) Quantification of N.Adi/Ma.Ar was measured through bone histomorphometry analysis of the selected area shown in (a) by Image-Pro Plus. N.Adi/Ma.Ar: number of adipocytes/the area of the bone marrow. Data represented as mean ± SD, *n* = 10. ^*∗*^*P* < 0.05; ^*∗∗∗∗*^*P* < 0.0001.

Microcomputed tomography (*μ*-CT) was used to analyze trabecular bone in the distal femur via different observation angles from different groups of mice (Figure 1(a)). When compared with sham-operated group, the data of trabecular bone in the distal femoral metaphyses from the OVX group showed that BV/TV, Tb.N, and Tb.Th decreased significantly; on the contrary, Tb.Sp was dramatically increased (Figure 1(b)). In a group of OVX mice treated with icariin (OVX + ICA), the measured parameters showed that intervention of icariin significantly attenuated the bone loss induced by ovariectomy. These results were consistent with the outcomes of tissue sections (Figure 2(a)). Sample sections from OVX mice demonstrated a paucity of cancellous bone (Figure 2(a)). Icariin treatment in OVX mice resulted in an increase in bone density. In the selective area of the distal femur from different groups, the number of adipocytes (N.Adi/Ma.Ar) in OVX mice distinctly increased compared with sham-operated control mice, whereas icariin treatment significantly decreased formation of adipocytes (Figure 2(b)).

### 3.2. Icariin Stimulates BMMSCs Proliferation and Osteogenic Differentiation and Inhibits Adipogenic Differentiation

**Figure 3:**
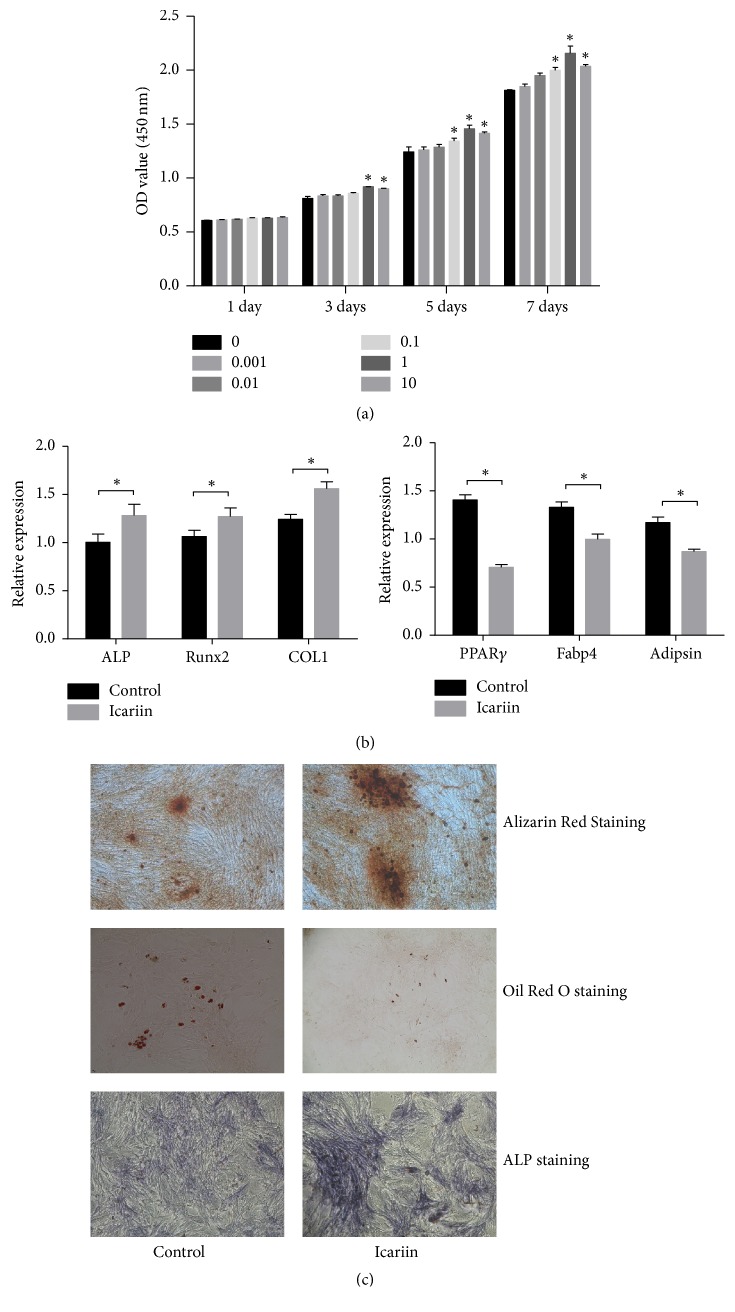
Icariin promotes osteogenesis differentiation and inhibits adipogenesis differentiation of BMMSCs. (a) Effect of icariin treatment on proliferation of BMMSCs at different time points. Data presented as mean ± SD. *n* = 3. ^*∗*^*P* < 0.05 versus control. (b) Effect of icariin on ALP activity and formation of calcium nodules and lipid droplets. (c) Effect of icariin on mRNA expression of ALP, Runx2, COL1, PPAR*γ*, Fabp4, and adipsin. Data presented as mean ± SD, *n* = 3.

Through CCK-8 kit, we found that no differences in cell numbers were observed in the concentrations of 0.001 and 0.01 *μ*M. The other concentrations of icariin showed a proliferative role on BMMSCs, but its proliferative function started at a different time point. The concentration of 1 *μ*M icariin showed the most proliferative effect and initiated its proliferative effect earlier (Figure 3(a)). 1 *μ*M icariin was selected in a later experiment. By intervention of icariin, icariin could upregulate the relative expression of related osteogenic genes—Runx2, ALP, and Collagen I—and downregulate the relative expression of related adipogenic genes—PPAR*γ*, Fabp4, and adipsin (Figure 3(c)). These selective genes—Runx2, ALP, Collagen I, PPAR*γ*, Fabp4, and adipsin—have been shown to have a proven function of regulating the differentiation of BMMSCs in previous studies [5]. In the morphological observation of osteogenesis, the number of Alizarin Red S stained nodules formed and the ALP expressed in the culture medium of icariin treatment were higher than in control. In adipogenesis, compared with the control, the formation of lipid droplets in the culture medium of icariin treatment was decreased (Figure 3(b)).

### 3.3. Participation of Canonical Wnt Signaling Pathway in BMMSCs Differentiation Intervened in by Icariin

**Figure 4:**
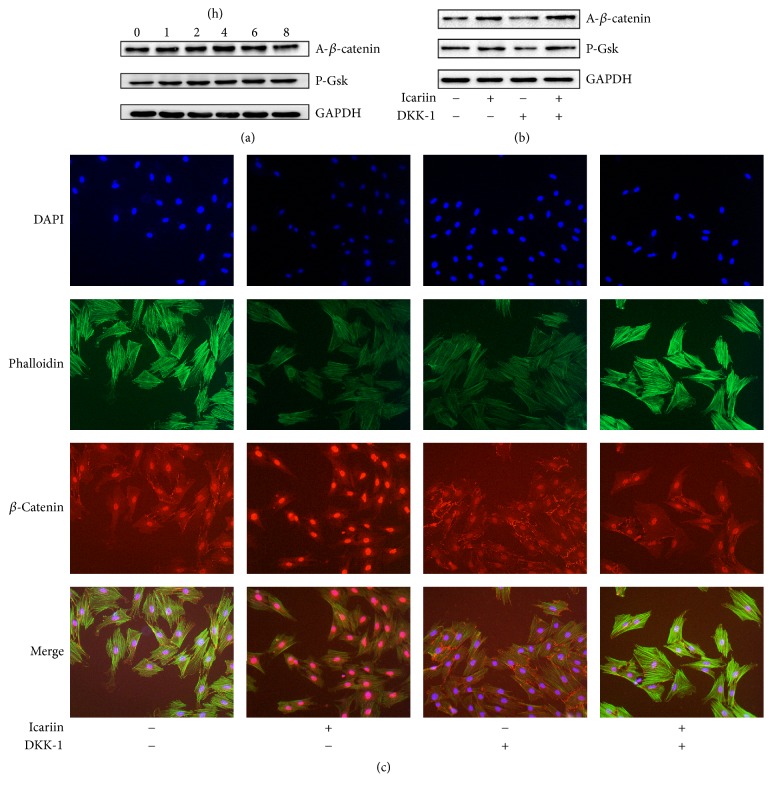
Icariin activated Wnt signaling pathway in BMMSCs. (a) Images of icariin intervention on expression of P-Gsk3*β* and *β*-catenin. (b) Images of icariin and DDK-1 intervention on expression of P-Gsk3*β* and *β*-catenin. (c) Immunofluorescence images of icariin and DKK-1 intervention on intracellular localization of *β*-catenin.

Wnt signaling pathway is an important pathway in cell fate determination and organogenesis. Therefore, we detected whether BMMSCs treated with icariin could activate canonical Wnt signaling pathway. BMMSCs were treated with 1 *μ*M icariin at different time points. Western blot showed that intervention of icariin promoted the phosphorylation of GSK-3*β* and increased the quantity of Non-phospho (Active) *β*-catenin. The peak phosphorylation level of GSK-3*β* appeared at 4 h. *β*-Catenin activation occurred at 1 h and reached the maximum at 2 h (Figure 4(a)). After BMMSCs were treated with icariin for 8 h, immunofluorescence showed that icariin facilitated transportation of *β*-catenin into the nucleus and more *β*-catenin appeared in the nucleus other than the cytoplasm compared with the control (Figure 4(c)).

### 3.4. Role of Canonical Wnt Signaling Pathway in Osteogenic and Adipogenic Differentiation during Icariin Treatment

**Figure 5:**
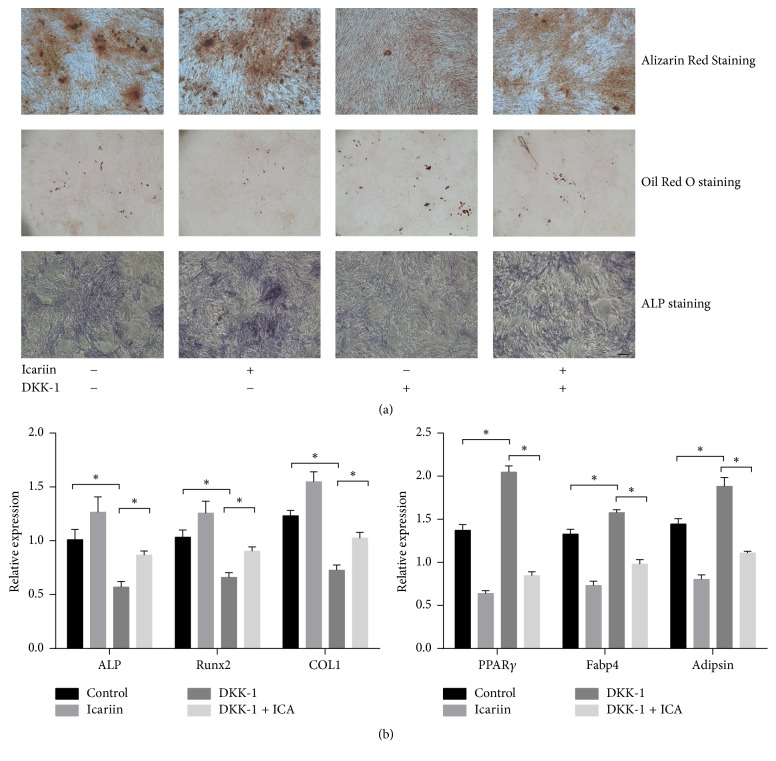
Icariin had an antagonistic effect on DDK-1 in the differentiation of BMMSCs. (a) Effect of icariin and DKK-1 intervention on ALP activity and formation of calcium nodules and lipid droplets. Scale bar: 100x magnification. (b) Effect of icariin and DKK-1 intervention on mRNA expression of ALP, Runx2, COL1, PPAR*γ*, Fabp4, and adipsin. Data presented as mean ± SD, *n* = 3. ^*∗*^*P* < 0.05.

DKK-1, a specific inhibitor of Wnt signaling pathway, can downregulate the phosphorylation of GSK-3*β* and promote degradation of *β*-catenin, so the quantity of *β*-catenin that is transported into the nucleus is reduced in immunofluorescence (Figures 4(b) and 4(c)). DKK-1 downregulated the relative expression of related osteogenic genes and upregulated the relative expression of related adipogenic genes (Figure 5(b)). During osteogenesis induction, the number of Alizarin Red S stained nodules and expression of ALP were significantly reduced by treatment of DKK-1; on the other hand, the treatment of DKK-1 in adipogenesis induction had a function of promoting the formation of lipid droplets (Figure 5(a)). When cells were treated with a combination of icariin and DKK-1, the downregulated phosphorylation of GSK-3*β* by treatment of DKK-1 solely was attenuated and the expression of *β*-catenin showed upregulation (Figure 4(b)). In detection of immunofluorescence, nuclear import of *β*-catenin showed an increase in combined intervention (Figure 4(c)). In the group with combined intervention, the relative expression of related adipogenic genes showed a significant difference compared with groups of control and DDK-1 intervention. However, not all relative expression of related osteogenic genes showed a significant difference compared with groups of control and DKK-1 intervention. Collagen I showed a difference compared with groups of control and DDK-1 intervention, but ALP and Runx2 only showed a difference compared with DKK-1 group (Figure 5(b)). In the induction culture process, the combined intervention of icariin and DKK-1 contributed to the reduced formation of lipid droplet and improved the formation of Alizarin Red S stained nodules and activity of ALP (Figure 5(a)).

## 4. Discussion

Osteoporosis had become a major burden for healthcare and individual and society costs, affecting a large population worldwide. Bone marrow plays an important role in maintaining bone homeostasis [39]. The different cells in the bone marrow can establish a functional relationship through locally produced soluble factors, extracellular matrix components, and systemic factors, and this connection contributes to a distinctive microenvironment [40, 41].

In normal condition, adipocytes within the bone marrow mainly derive from adipogenic differentiation of BMMSCs, and other studies showed that, in some pathological processes of skeletal disorders, such as osteoporosis, BMMSCs-derived osteoblasts and chondrocytes had the ability to transdifferentiate into adipocytes [42, 43]. The increased adipocyte formation in the bone marrow had been linked to increased fracture risk and reduced bone density, and this effect was considered even more pronounceable in osteoporosis [44–46]. Further research on BMMSC differentiation showed that the upregulation of PPAR*γ* positively stimulates adipocyte differentiation while serving as a dominant negative stimulus for osteogenic differentiation [47, 48]. Administration of rosiglitazone (a PPAR*γ* agonist) in mice showed bone loss and reduced bone formation with increased adipocyte accumulation [49, 50]. In contrast, in heterozygous deficient animals, a PPAR*γ* deficient mouse model showed increased bone mass density and osteoblastogenesis [51]. Additionally, Runx2^−/−^ calvarial cells spontaneously showed adipocyte differentiation, which suggested that Runx2 expression in BMMSCs may inhibit the BMMSCs differentiation into adipocytes [52].

In the bone marrow, there are two kinds of stem cells—hematologic stem cells and nonhematopoietic stem cells—and mesenchymal stem cells belong to the latter. Mesenchymal stem cells differentiate into osteoblast and osteoclast derived from hematologic stem cells. Osteoblast-mediated bone formation and osteoclast-mediated bone resorption are engaged in an unceasing process of bone reconstruction and determine bone mass and bone quality. Initially, cell researches on the occurrence of osteoporosis were concentrated on osteoclastic activity with its bone resorption processes or solely osteoblastic differentiation of BMMSCs and icariin had been proven its function on the above-mentioned aspects [53–56]. BMMSCs' own ability to undertake osteogenic differentiation and adipogenic differentiation and adipose tissue within the bone marrow has been demonstrated to have a relationship with bone homeostasis. When 12-week-old mice underwent ovariectomy, the images of the distal femur showed sharply reduced cancellous bone which is consistent with previous studies [57, 58]. Moreover, we focused on the accumulation of adipose tissue in the bone marrow, and thus we found that the bone marrow of mice with ovariectomy was infiltrated with adipocytes, which is in accordance with clinical studies [59, 60]. OVX mice treated with icariin not only had mitigated bone loss but also had alleviated adipocyte accumulation. The detailed quantitative parameter obtained from micro-CT and tissue slice also showed consistent outcomes.

In an in vitro study, we firstly investigate the proper concentration of icariin to intervene in its effect on BMMSCs proliferation and differentiation. In previous researches, the concentration of icariin at 0.1 *μ*M could promote the proliferation of BMMSCs [61]. We demonstrated that the concentration of icariin lower than 1 *μ*M was optimal in improving BMMSCs proliferation and regulating differentiation. In our present study, 1 *μ*M icariin intervention could regulate the differentiation of BMMSCs toward osteoblast lineage and away from adipocyte lineage from morphological exhibition. At the genetic level, icariin upregulated osteogenic related genes and downregulated adipogenic related genes at different levels. Hasan et al. reported that diosmin could attenuate fibrosis of hepatic stellate cells via activating Wnt signaling pathway [62]. Hesperetin was demonstrated to elicit *β*-catenin-dependent osteoblastic differentiation of periodontal ligament stem cells in high glucose condition [63]. Another research also reported that Wnt pathway is critical for naringin to counterbalance the negative effect on osteogenesis of adipose-derived mesenchymal stem cells in oxidative stress [64]. Diosmin, hesperetin, naringin, and icariin are part of bioflavonoids, so we detected the influence of icariin on Wnt pathway. In the following study, icariin could upregulate the phosphorylation of GSK-3*β* in N-terminal Ser9 site. The activity of phosphorylated GSK-3*β* is in an inhibitory state and the intracellular depolymerized complex is unable bind to the *β*-catenin in the cytoplasm [65]. Therefore, the nonphosphorylated state of *β*-catenin was upregulated and the nonphosphorylated *β*-catenin entered the nucleus. We then utilized DKK-1, an inhibitory secretory glycoprotein for Wnt signaling pathway, to investigate whether icariin regulates the bidirectional differentiation of BMMSCs through Wnt/*β*-catenin signaling pathway. We realized that DKK-1 obviously blocked upregulated ALP expression and reduced the deposition of extracellular calcified nodules compared with the control group. The osteogenic related genes showed reduced expression with DKK-1 treatment. In aspect of adipogenic differentiation, DKK-1 significantly increased accumulation of lipid droplets and expression of adipogenic related genes. In accordance with expectation, icariin neutralized the effect of DKK-1 on the bidirectional differentiation of BMMSCs in morphology and gene level. The reduced phosphorylation of GSK-3*β* and expression of nonphosphorylated *β*-catenin induced by DKK-1 were counterbalanced by icariin, with less *β*-catenin anchored in the cell membrane and more *β*-catenin transferred into the nucleus.

In conclusion, this study demonstrated the ability of icariin to regulate the bidirectional differentiation of BMMSCs; with the use of DDK-1, the Wnt signaling pathway inhibitor, we confirmed that this regulatory effect of icariin is achieved by canonical Wnt signaling pathway.

## Figures and Tables

**Table 1:** List of primers used in quantitative real-time RT-PCR.

Gene	Forward (5′-3′)	Reverse (5′-3′)
ALP	AGGGTGGGTTTCTCTCTTGG	AGAGAAGGGGTAGGGGAGAG
Runx2	GGGACCGACACAGCCATATA	TCTTAGGGTCTCGGAGGGAA
COL1	TCAAGATGGTGGCCGTTACT	CATCTTGAGGTCACGGCATG
PPAR*γ*	GGAATCAGCTCTGTGGACCT	TCAGCTCTTGTGAACGGGAT
Fabp4	ATGTGCAGAAGTGGGATGGA	TGCAAATTTCAGTCCAGGGC
Adipsin	AGTGTCAATCATGGACCGGA	ACTCGAGATCCCCACGTAAC
GAPDH	CAAGTTCAACGGCACAGTCA	CCCCATTTGATGTTAGCGGG

## References

[1] Menagh P. J., Turner R. T., Jump D. B. (2010). Growth hormone regulates the balance between bone formation and bone marrow adiposity. *Journal of Bone and Mineral Research*.

[2] Verma S., Rajaratnam J. H., Denton J., Hoyland J. A., Byers R. J. (2002). Adipocytic proportion of bone marrow is inversely related to bone formation in osteoporosis. *Journal of Clinical Pathology*.

[3] Qiu W., Andersen T. E., Bollerslev J., Mandrup S., Abdallah B. M., Kassem M. (2007). Patients with high bone mass phenotype exhibit enhanced osteoblast differentiation and inhibition of adipogenesis of human mesenchymal stem cells. *Journal of Bone and Mineral Research*.

[4] Wu L., Zhao X., He B., Jiang J., Xie X.-J., Liu L. (2016). The possible roles of biological bone constructed with peripheral blood derived epcs and bmscs in osteogenesis and angiogenesis. *BioMed Research International*.

[5] Li W., Li G., Zhang Y. (2015). Role of P2×7 receptor in the differentiation of bone marrow stromal cells into osteoblasts and adipocytes. *Experimental Cell Research*.

[6] Jiang Y., Jahagirdar B. N., Reinhardt R. L. (2002). Pluripotency of mesenchymal stem cells derived from adult marrow. *Nature*.

[7] Backesjo C. M., Li Y., Lindgren U., Haldosen L. A. (2008). Activation of Sirt1 decreases adipocyte formation during osteoblast differentiation of mesenchymal stem cells. *Cells Tissues Organs*.

[8] Guntur A. R., Kawai M., Le P. (2011). An essential role for the circadian-regulated gene Nocturnin in osteogenesis: The importance of local timekeeping in skeletal homeostasis. *Annals of the New York Academy of Sciences*.

[9] Idris A. I., Sophocleous A., Landao-Bassonga E. (2009). Cannabinoid Receptor Type 1 Protects against Age- Related Osteoporosis by Regulating Osteoblast and Adipocyte Differentiation in Marrow Stromal Cells. *Cell Metabolism*.

[10] Sato K., Mera H., Wakitani S., Takagi M. (2017). Effect of epigallocatechin-3-gallate on the increase in type II collagen accumulation in cartilage-like MSC sheets. *Bioscience, Biotechnology, and Biochemistry*.

[11] Bateman M. E., Strong A. L., Hunter R. S. (2017). Osteoinductive effects of glyceollins on adult mesenchymal stromal/stem cells from adipose tissue and bone marrow. *Phytomedicine*.

[12] Wu T., Shu T., Kang L. (2017). Icaritin, a novel plant-derived osteoinductive agent, enhances the osteogenic differentiation of human bone marrow- and human adipose tissue-derived mesenchymal stem cells. *International Journal of Molecular Medicine*.

[13] Casado-Diaz A., Tunez-Finana I., Mata-Granados J. M. (2017). Serum from postmenopausal women treated with a by-product of olive-oil extraction process stimulates osteoblastogenesis and inhibits adipogenesis in human mesenchymal stem-cells (MSC). *Experimental Gerontology*.

[14] Zhou Z., Xu Z., Wang F. (2016). New strategy to rescue the inhibition of osteogenesis of human bone marrow-derived mesenchymal stem cells under oxidative stress: Combination of vitamin C and graphene foams. *Oncotarget *.

[15] Cao J., Ma Y., Yao W., Zhang X., Wu D. (2017). Retinoids Regulate Adipogenesis Involving the TGF*β*/SMAD and Wnt/*β*-Catenin Pathways in Human Bone Marrow Mesenchymal Stem Cells. *International Journal of Molecular Sciences*.

[16] Maurin A. C., Chavassieux P. M., Frappart L., Delmas P. D., Serre C. M., Meunier P. J. (2000). Influence of mature adipocytes on osteoblast proliferation in human primary cocultures. *Bone*.

[17] Wan Y., Chong L.-W., Evans R. M. (2007). PPAR-*γ* regulates osteoclastogenesis in mice. *Nature Medicine*.

[18] Zhang G., Qin L., Shi Y. (2007). Epimedium-derived phytoestrogen flavonoids exert beneficial effect on preventing bone loss in late postmenopausal women: A 24-month randomized, double-blind and placebo-controlled trial. *Journal of Bone and Mineral Research*.

[19] Huang J., Yuan L., Wang X., Zhang T.-L., Wang K. (2007). Icaritin and its glycosides enhance osteoblastic, but suppress osteoclastic, differentiation and activity in vitro. *Life Sciences*.

[20] Bian Q., Huang J. H., Liu S. F. (2012). Different molecular targets of Icariin on bMSCs in CORT and OVX -rats. *Frontiers in Bioscience*.

[21] Wu Y., Xia L., Zhou Y., Xu Y., Jiang X. (2015). Icariin induces osteogenic differentiation of bone mesenchymal stem cells in a MAPK-dependent manner. *Cell Proliferation*.

[22] Ye Y., Jing X., Li N., Wu Y., Li B., Xu T. (2017). Icariin promotes proliferation and osteogenic differentiation of rat adipose-derived stem cells by activating the RhoA-TAZ signaling pathway. *Biomedicine & Pharmacotherapy*.

[23] Wang P., Zhang F., He Q. (2016). Flavonoid compound icariin activates hypoxia inducible factor-1*α* in chondrocytes and promotes articular cartilage repair. *PLoS ONE*.

[24] Hsieh T.-P., Sheu S.-Y., Sun J.-S., Chen M.-H. (2011). Icariin inhibits osteoclast differentiation and bone resorption by suppression of MAPKs/NF-*κ*B regulated HIF-1*α* and PGE_2_ synthesis. *Phytomedicine*.

[25] Chung B.-H., Kim J.-D., Kim C.-K. (2009). Icariin stimulates angiogenesis by activating the MEK/ERK- and PI3K/Akt/eNOS-dependent signal pathways in human endothelial cells. *Biochemical and Biophysical Research Communications*.

[26] Ye C., Zhang W., Wang S. (2016). Icariin Promotes Tendon-Bone Healing during Repair of Rotator Cuff Tears: A Biomechanical and Histological Study. *International Journal of Molecular Sciences*.

[27] Lu Y.-F., Xu Y.-Y., Jin F., Wu Q., Shi J.-S., Liu J. (2014). Icariin is a PPAR*α* activator inducing lipid metabolic gene expression in mice. *Molecules*.

[28] Zheng Z.-G., Zhou Y.-P., Zhang X. (2016). Anhydroicaritin improves diet-induced obesity and hyperlipidemia and alleviates insulin resistance by suppressing SREBPs activation. *Biochemical Pharmacology*.

[29] Li H., Yuan Y., Zhang Y., Zhang X., Gao L., Xu R. (2017). Icariin inhibits AMPK-dependent autophagy and adipogenesis in adipocytes In Vitro and in a model of graves' orbitopathy In Vivo. *Frontiers in Physiology*.

[30] MacDonald B. T., Tamai K., He X. (2009). Wnt/*β*-catenin signaling: components, mechanisms, and diseases. *Developmental Cell*.

[31] Logan C. Y., Nusse R. (2004). The Wnt signaling pathway in development and disease. *Annual Review of Cell and Developmental Biology*.

[32] Little R. D., Recker R. R., Johnson M. L. (2002). High bone density due to a mutation in LDL-receptor-related protein. *The New England Journal of Medicine*.

[33] Poole K. E. S., Van Bezooijen R. L., Loveridge N. (2005). Sclerostin is a delayed secreted product of osteocytes that inhibits bone formation. *The FASEB Journal*.

[34] Baron R., Kneissel M. (2013). WNT signaling in bone homeostasis and disease: from human mutations to treatments. *Nature Medicine*.

[35] Vanella L., Sodhi K., Kim D. H. (2013). Increased heme-oxygenase 1 expression in mesenchymal stem cell-derived adipocytes decreases differentiation and lipid accumulation via upregulation of the canonical Wnt signaling cascade. *Stem Cell Research & Therapy*.

[36] Sreejit P., Dilip K. B., Verma R. S. (2012). Generation of mesenchymal stem cell lines from murine bone marrow. *Cell and Tissue Research*.

[37] Weng T., Xie Y., Huang J. (2014). Inactivation of Vhl in osteochondral progenitor cells causes high bone mass phenotype and protects against age-related bone loss in adult mice. *Journal of Bone and Mineral Research*.

[38] Bouxsein M. L., Boyd S. K., Christiansen B. A., Guldberg R. E., Jepsen K. J., Müller R. (2010). Guidelines for assessment of bone microstructure in rodents using micro-computed tomography. *Journal of Bone and Mineral Research*.

[39] Pino A. M., Rosen C. J., Rodríguez J. P. (2012). In Osteoporosis, differentiation of mesenchymal stem cells (MSCs) improves bone marrow adipogenesis. *Biological Research*.

[40] Raisz L. G. (2005). Pathogenesis of osteoporosis: concepts, conflicts, and prospects. *The Journal of Clinical Investigation*.

[41] Sambrook P., Cooper C. (2006). Osteoporosis. *The Lancet*.

[42] Song L., Tuan R. S. (2004). Transdifferentiation potential of human mesenchymal stem cells derived from bone marrow. *The FASEB Journal*.

[43] Gao B., Huang Q., Lin Y. S. (2014). Dose-dependent effect of estrogen suppresses the osteo-adipogenic transdifferentiation of osteoblasts via canonical Wnt signaling pathway. *PLoS One*.

[44] Chen X.-D., Shi S., Xu T., Robey P. G., Young M. F. (2002). Age-related osteoporosis in biglycan-deficient mice is related to defects in bone marrow stromal cells. *Journal of Bone and Mineral Research*.

[45] Carbonare L. D., Valenti M. T., Zanatta M., Donatelli L., Lo Cascio V. (2009). Circulating mesenchymal stem cells with abnormal osteogenic differentiation in patients with osteoporosis. *Arthritis & Rheumatology*.

[46] Egermann M., Heil P., Tami A. (2010). Influence of defective bone marrow osteogenesis on fracture repair in an experimental model of senile osteoporosis. *Journal of Orthopaedic Research*.

[47] Ge C., Cawthorn W. P., Li Y., Zhao G., Macdougald O. A., Franceschi R. T. (2016). Reciprocal control of osteogenic and adipogenic differentiation by ERK/MAP kinase phosphorylation of Runx2 and PPAR*γ* transcription factors. *Journal of Cellular Physiology*.

[48] Tseng K.-Y., Lin S. (2015). Zinc finger factor 521 enhances adipogenic differentiation of mouse multipotent cells and human bone marrow mesenchymal stem cells. *Oncotarget *.

[49] Ali A. A., Weinstein R. S., Stewart S. A., Parfitt A. M., Manolagas S. C., Jilka R. L. (2005). Rosiglitazone causes bone loss in mice by suppressing osteoblast differentiation and bone formation. *Endocrinology*.

[50] Tang X.-L., Wang C.-N., Zhu X.-Y., Ni X. (2015). Rosiglitazone inhibition of calvaria-derived osteoblast differentiation is through both of PPAR*γ* and GPR40 and GSK3*β*-dependent pathway. *Molecular and Cellular Endocrinology*.

[51] Cock T.-A., Back J., Elefteriou F. (2004). Enhanced bone formation in lipodystrophic PPAR*γ*hyp/hyp mice relocates haematopoiesis to the spleen. *EMBO Reports*.

[52] Kobayashi H., Gao Y.-H., Ueta C., Yamaguchi A., Komori T. (2000). Multilineage differentiation of Cbfa1-deficient calvarial cells in vitro. *Biochemical and Biophysical Research Communications*.

[53] Wu Z., Ou L., Wang C. (2017). Icaritin induces MC3T3-E1 subclone14 cell differentiation through estrogen receptor-mediated ERK1/2 and p38 signaling activation. *Biomedicine & Pharmacotherapy*.

[54] Hsiao H., Wu J., Lin W. (2017). (−)-Epicatechin 3-O-*β*-d-allopyranoside prevent ovariectomy-induced bone loss in mice by suppressing RANKL-induced NF-*κ*B and NFATc-1 signaling pathways. *BMC Complementary and Alternative Medicine*.

[55] Huang Y.-L., Shen C.-C., Shen Y.-C., Chiou W.-F., Chen C.-C. (2017). Anti-inflammatory and Antiosteoporosis Flavonoids from the Rhizomes of Helminthostachys zeylanica. *Journal of Natural Products*.

[56] Song S.-H., Zhai Y.-K., Li C.-Q. (2016). Effects of total flavonoids from Drynariae Rhizoma prevent bone loss in vivo and in vitro. *Bone Reports*.

[57] Zhang Y., Guan H., Li J., Fang Z., Chen W., Li F. (2015). Amlexanox suppresses osteoclastogenesis and prevents ovariectomy-induced bone loss. *Scientific Reports*.

[58] Guan H., Zhao L., Cao H., Chen A., Xiao J. (2015). Epoxyeicosanoids suppress osteoclastogenesis and prevent ovariectomy-induced bone loss. *The FASEB Journal*.

[59] Blake G. M., Griffith J. F., Yeung D. K. W., Leung P. C., Fogelman I. (2009). Effect of increasing vertebral marrow fat content on BMD measurement, T-Score status and fracture risk prediction by DXA. *Bone*.

[60] Griffith J. F., Yeung D. K. W., Antonio G. E. (2005). Vertebral bone mineral density, marrow perfusion, and fat content in healthy men and men with osteoporosis: dynamic contrast-enhanced MR imaging and MR spectroscopy. *Radiology*.

[61] Wei Q., He M., Chen M. (2017). Icariin stimulates osteogenic differentiation of rat bone marrow stromal stem cells by increasing TAZ expression. *Biomedicine & Pharmacotherapy*.

[62] Hasan H. F., Abdel-Rafei M. K., Galal S. M. (2017). Diosmin attenuates radiation-induced hepatic fibrosis by boosting PPAR-*γ* expression and hampering miR-17-5p-activated canonical Wnt–*β*-catenin signaling. *The International Journal of Biochemistry & Cell Biology*.

[63] Kim S. Y., Lee J.-Y., Park Y.-D., Kang K. L., Lee J.-C., Heo J. S. (2013). Hesperetin alleviates the inhibitory effects of high glucose on the osteoblastic differentiation of periodontal ligament stem cells. *PLoS ONE*.

[64] Wang L., Zhang Y.-G., Wang X.-M., Ma L.-F., Zhang Y.-M. (2015). Naringin protects human adipose-derived mesenchymal stem cells against hydrogen peroxide-induced inhibition of osteogenic differentiation. *Chemico-Biological Interactions*.

[65] Yan Y., Li G., Tian X. (2015). Ischemic preconditioning increases GSK-3*β*/*β*-catenin levels and ameliorates liver ischemia/reperfusion injury in rats. *International Journal of Molecular Medicine*.

